# Individual quality explains association between plumage colouration, arrival dates and mate acquisition in yellow warblers (*Setophaga petechia*)

**DOI:** 10.1186/1472-6785-14-13

**Published:** 2014-05-07

**Authors:** Teri B Jones, Anna Drake, David J Green

**Affiliations:** 1 Centre for Wildlife Ecology, Department of Biological Sciences, Simon Fraser University, Burnaby, BC V5A 1S6, Canada

**Keywords:** Carry-over effects, Seasonal interactions, Breeding phenology, Plumage colour, Carotenoid-based colouration, Yellow warbler, American redstart

## Abstract

**Background:**

In many bird species colour traits influence social dominance and breeding success. In our study we first evaluated whether the colour of the basic plumage (tail feathers grown at the end of the breeding season), that provides an index of individual quality, influenced winter habitat use by yellow warblers. We then evaluated whether winter habitat use (inferred using δ^13^C and δ^15^N signatures of winter grown greater-coverts) influenced alternate plumage colouration, after controlling for individual quality using basic plumage colouration. Finally, we investigated whether basic and alternate plumage colouration influenced arrival dates, mate acquisition, breeding phenology and reproductive success of yellow warblers breeding in southern (Revelstoke, B.C.) and arctic (Inuvik, N.W.T.) Canada.

**Results:**

The colour (chroma and hue) of tail feathers, grown on the breeding grounds, was not related to subsequent winter habitat use. Greater covert and tail feather colour (chroma and hue) were correlated, suggesting genetics and/or individual quality played a role in pigment deposition. After controlling for individual difference in tail colour, δ^13^C values did not explain any variation in greater covert colour, but birds with high δ^15^N signatures had greater coverts with higher chroma. Male arrival dates varied with tail chroma in Revelstoke and tail hue in Inuvik. Males that arrived early paired with older and/or more colourful mates that initiated clutches earlier, and at one site (Revelstoke) were more likely to fledge young. In addition, in Revelstoke (but not Inuvik) males with high tail hue also acquired more colourful mates. In contrast, after controlling for individual differences in tail colour, greater covert colour did not affect male arrival date, the quality of the mate obtained or reproductive success in either population.

**Conclusions:**

Our results suggest that plumage colour effects on breeding phenology and mate acquisition result from differences in the intrinsic quality of individuals rather than a carry-over effect of winter habitat use.

## Background

Carry-over effects, where events or processes in one season affect the performance of an individual in a subsequent season, have been argued to be a widespread phenomena with the potential to influence both individual fitness and population dynamics [1]. In migratory birds, for example, individuals with access to high quality winter habitat may be in better condition at the end of the winter period [2,3], depart on migration [4,5] and arrive on the breeding grounds earlier [6,7] and as a consequence have higher reproductive success [8-10]. In this example, the relationship between winter habitat quality and subsequent reproductive success may arise because high quality winter habitat confers a fitness advantage to individuals (extrinsic factors) or because high quality winter habitat is accessed by high quality individuals who perform well throughout the annual cycle (intrinsic factors). Our ability to disentangle the roles of extrinsic and intrinsic factors in driving carryover effects is undermined by the difficulty of manipulating these factors independently in wild populations [1], but see [4].

**Figure 1:**
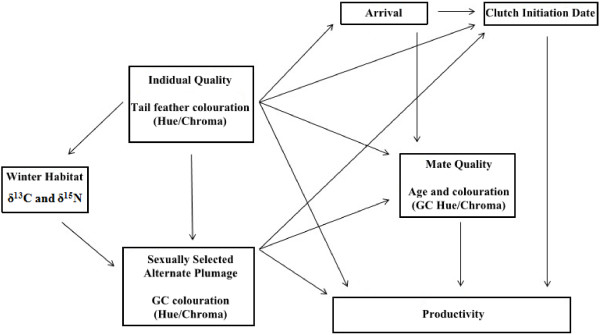
**Potential pathways for seasonal interactions.** Summary of possible pathways for seasonal interactions mediated through sexually selected carotenoid plumage colouration of feathers grown on the wintering grounds (greater covert chroma and hue) and individual quality (as measured through plumage coluration of tail feathers grown on shared breeding grounds).

Carry-over effects associated with access to high quality winter habitat may be generated because winter habitat influences migration and breeding phenology (see refs above), but could also arise because winter habitat conditions influence sexually selected plumage ornamentation involved in inter- and intra-sexual interactions on the breeding grounds (e.g. [11]). Study of the relationships between sexually selected plumage, winter habitat use, and breeding performance in birds that undergo moults on both breeding and wintering grounds may provide an opportunity to evaluate the relative importance of extrinsic and intrinsic factors in driving carryover effects. In these species, the colour of basic (“non-breeding”) plumage, grown on shared breeding grounds at the end of the breeding season, provides an index of individual quality that is largely independent of variation in breeding habitat [12,13]. In contrast, the colour of alternate (“breeding”) plumage grown on the wintering grounds is likely to reflect both individual quality and the resources available in the winter habitat occupied. The importance of extrinsic factors to carry-over effects can then be assessed by investigating how the colour of alternate plumage influences individual breeding performance after controlling for the colour of their basic plumage (Figure 1).

Carotenoid pigments deposited in feathers contribute to the red, orange and yellow plumage of many bird species (e.g. house finch *Haemorhous mexicanus*[14]; American redstart *Setophaga ruticilla*[15]; American goldfinch *Spinus tristis*[16]). Carotenoids are unique in that they cannot be synthesised *de novo* in the body [15] and therefore have to be ingested with food. Carotenoid-based plumage colouration is known to be affected by access to dietary carotenoids (e.g. [14,17]). Carotenoids also reflect immune responses to parasite load (e.g. [18]) and may play an important role as antioxidants [19], although their relative importance compared with other antioxidants is under considerable debate see [20]. The degree of carotenoid-based colouration in plumage may therefore be an indicator of both the compound’s availability in the environment and an individual’s ability to dedicate it to pigmentation, rather than other functions [21,22]. Colourful individuals may therefore be expected to occupy superior habitat [23] and/or be in superior condition [12,13,24].

Yellow warblers *Setophaga petechia* are neotropical migrants that undergo two moults per year: a complete prebasic molt on the breeding grounds prior to fall migration, and a partial prealternate molt on the wintering grounds where they replace contour feathers and some or all of their greater coverts and tertials [25,26]. The yellow pigmentation of both basic and alternate plumage in this species is produced by a single carotenoid pigment, lutein [27]. Plasma and feather lutein concentration has previously been shown to be positively correlated with feather colour (chroma) in adult American goldfinches [16] and nestling great tits, *Parus major*[17]. In this study we examined the relationship between the yellow colour (chroma and hue) of basic and alternate plumage (tail and greater covert feathers, respectively), winter habitat use, and breeding performance of yellow warblers in British Columbia and the Northwest Territories of Canada.

We first examined whether the colour of the basic plumage grown on the breeding grounds (tail feathers) influenced winter habitat use, inferred using stable isotope signatures (δ^13^C and δ^15^N) in alternate plumage. We then assessed whether winter habitat use influenced the colour of the alternate plumage (greater coverts) grown on the wintering grounds, after controlling for individual variation in basic plumage colouration. Finally, we investigated whether the colour of the basic and alternate plumage influenced arrival dates, mate acquisition, breeding phenology and reproductive success. We predicted that (1) individuals with more colourful basic plumage would acquire higher quality (more mesic) wintering habitats (2) individuals from high quality winter habitats would have more colourful alternate plumage and (3) more colourful males would arrive earlier, pair with older and more colourful mates, and have greater reproductive success. Evidence that winter habitat use influences the colour of an individual’s alternate plumage, and their subsequent breeding performance, after controlling for the colour of their basic plumage would indicate the importance of extrinsic factors in driving carryover effects.

## Methods

### Study species and locations

Yellow warblers are small (9–11 g) migratory songbirds that breed throughout North America and over-winter in Mexico, Central America, and in northern regions of South America [28]. Western and eastern breeding populations are partially segregated on the wintering grounds, with western populations over-wintering in Mexico and Central America, while eastern populations over-winter in Central and South America [29]. Wintering yellow warblers often maintain foraging territories and display interspecies aggression [28,30,31]. In Mexico yellow warblers have clearly defined home ranges, respond with aggression to conspecific playback [32] and show evidence of winter site fidelity [Valdez Juarez, unpublished data]. This species breeds in riparian and wet deciduous habitat, and overwinters in a variety of habitat types, ranging from riparian forests, swamp and mangrove to dry coastal scrub, and agricultural areas including pastures and sun/semi-shade coffee plantations [10,30,33]. Drake [32] also found that some winter habitat types (riparian and agricultural), but not others (scrub) are preferentially occupied by older males, suggesting that winter habitat varies in quality.

Between 2009 and 2011, we collected feather samples and reproductive data from two populations of Yellow Warblers breeding in western Canada. In Revelstoke, British Columbia (50° 57’N, 118° 10’ W; hereafter Revelstoke) pairs were monitored at three sites of approximately 30 ha along the Upper Arrow Lakes Reservoir (elevation 435–442 m). This habitat consisted of seasonally flooded grassland and riparian habitat dominated by willow shrubs (*Salix* spp.) and black cottonwood (*Populus trichocarpa*). In Inuvik, Northwest Territories (68° 21’ N, 133° 45’ W; hereafter Inuvik) pairs were monitored at one, 24 ha site located along the Mackenzie River (elevation 3–6 m). This habitat was dominated by willow shrubs (*Salix* spp.) and green alder (*Alnus crispa*) at elevations that experienced seasonal flooding during spring ice-break and white spruce (*Picea glauca*) intermixed with these species at elevations above the flood zone.

### Feather sampling

Breeding individuals were captured at both study locations through targeted mist-netting using audio playback. Males were generally captured within one to three days of their arrival on territories. Females were generally captured later in the season, often during nest building. Individuals were classified as yearlings (i.e. second year birds) or older birds (i.e. after-second year birds) based on the shape and colour of their primary coverts, the presence of a moult limit, and the shape and colour of their tail feathers [25]. Previously unbanded birds were marked with a combination of three colour bands and a Canadian Wildlife Service-issued aluminium band. We collected one tail feather (the third outer right rectrix) and three greater coverts feathers per individual, sampling a total of 119 individuals in Revelstoke (male = 66, female = 53) and 244 individuals in Inuvik (male = 130, female = 114). Yellow warblers are known to replace a variable number of greater coverts each year during their prealternate molt, and only newly replaced feathers (easily identifiable based on wear and colour) were collected for this study. Greater covert feathers were chosen over breast feathers to reflect winter grown colouration, as we have observed more variable isotopic signatures in breast feathers indicating that some may be moulted at different points in the year.

We determined the hue (colour) and chroma (purity of colour) of the yellow regions of greater covert and tail feathers from digital images using Adobe Photoshop CS6. An additional image depicts the colour properties hue and chroma more clearly [see Additional file 1]. Feathers were first scanned in colour at 1500 dpi with a yellow reference chip (hue = 40, chroma = 78). Three measurements were then collected from the yellow inner web of the tail feathers, the broad yellow edge of the greater coverts, and the reference chip using the eyedropper tool set to a 5x5 pixel sampling area. We then averaged the three measurements to produce single hue and chroma values for the basic and alternate plumage of each individual. To account for differences in the scans, hue and chroma measures were standardized against the standard reference chip, and these residual colour values were used in all analyses. Colour measures produced using similar methods have high repeatability (e.g. [17,24]) and are strongly correlated with spectrometer reflectance values [34]. The repeatability of hue and chroma measures in our study was high (Tail feathers: hue = 0.85, chroma = 0.94, n = 30; Greater coverts: hue = 0.88, chroma = 0.89, n = 30).

### Winter habitat use

We inferred aspects of winter habitat use using stable isotopes (δ^13^C and δ^15^N) signatures from winter-grown greater coverts. Feathers grown in wetter (mesic) habitats are expected to have lower δ^13^C levels than feathers grown in drier (xeric) habitats as δ^13^C in the diet varies with the photosynthetic pathways utilized by plants at the base of the food chain [35]. Consistent with this, the δ^13^C values of blood samples collected from yellow warblers captured in riparian habitat in Mexico are more depleted than δ^13^C ratios found in drier scrub and agricultural habitats [10], which, due to male bias observed in riparian suggests higher habitat quality [32]. Previous studies of American redstarts [4], and black-throated blue warblers (*Setophaga caerulescens*) [3] have also found that birds wintering in more mesic habitat had more depleted δ^13^C ratios and are in superior condition to birds from xeric scrub habitats, suggesting that mesic habitats are high quality winter habitats for warblers and that habitat quality can be evaluated using δ^13^C ratios. δ^15^N values are more difficult to interpret as they can vary with trophic level of dominant prey items [36], can be influenced by local landscape factors (e.g. agriculture), and, on a larger geographic scale, are also influenced by temperature and precipitation [37]. In yellow warblers, blood-δ^15^N is more enriched in the dry Pacific coast region of Mexico and more depleted in the wetter Gulf region of Mexico [10].

The isotopic analysis of greater covert samples was conducted at the University of California Davis Stable Isotope Facility (California, USA). Prior to analysis, feathers were washed in a 2:1 chloroform:methanol solution for 24 hours, and air-dried for a further 24 hours to remove remaining solvent. 1 ± 0.2 mg of tissue from each individual was analyzed using a PDZ Europa ANCA-GSL elemental analyzer interfaced to a PDZ Europa 20–20 isotope ratio mass spectrometer (Sercon Ltd., Cheshire, UK). Delta values are expressed relative to the international standards: Vienna PeeDee Belemnite (V-PDB) for δ^13^C and Air for δ^15^N. Estimated measurement precision is 0.2% for δ^13^C and 0.3% for δ^15^N.

### Breeding phenology and reproductive success

Study sites were monitored every one to three days beginning in early- and mid-May in Revelstoke and Inuvik, respectively. We recorded when male yellow warblers first arrived on a territory (“arrival date”), determined the identity of their mate, and followed pairs (30–50 pairs per season) throughout the entire breeding season. We located all nests by following females during nest-building or by conducting systematic searches of suitable nesting habitat. “Clutch initiation date” was defined as the date when females laid the first egg in their first nest of the season. These nests and all re-nesting attempts were checked every three days to determine clutch and brood size, the nest fate, and fledging success. The number of yellow warbler offspring fledged from a nest was assumed to equal the number of nestlings in the nest on day 7 (nestlings typically fledge on day 9), unless evidence of predation was observed. Fledging success was usually confirmed by locating the parents with some or all of their fledglings. Annual productivity for an individual was defined as the sum of all within-pair offspring successfully fledged over all nesting attempts of the season.

### Data analysis

We first examined whether tail and greater covert colour (hue and chroma) varied in the two breeding populations and described how feather colour varied with gender and age within the two populations. We also assessed whether there was a correlation between the colour (hue and chroma) of the tail and greater covert feathers within individuals and a correlation between the two measures of colour (hue and chroma) within either feather block.

We next examined whether the δ^13^C and δ^15^N signatures of greater coverts grown on the wintering grounds was related to the colour (hue and chroma) of an individual’s tail feather grown on the breeding grounds the previous summer. In this analysis we initially examined how δ^13^C or δ^15^N varied with sampling year, breeding population, and the gender and age of an individual. We did this by a starting with a full model that included all of these main effects and their two-way interactions and progressively dropped non-significant (p > 0.05) terms until only significant terms remained. We then asked whether adding tail colour (hue or chroma) explained any additional variance in δ^13^C and δ^15^N signatures. Hue and chroma measures were added independently as the two measures were correlated (see results). Significance was assessed using an F-test associated with the effect of dropping terms from a more complex model including all significant terms and the term of interest.

We next evaluated whether the colour (hue or chroma) of the greater coverts varied with winter habitat use, inferred from the δ^13^C and δ^15^N ratios of the same feathers. We initially fitted full models that included all main effects; breeding population, gender, age, tail hue or tail chroma and two-way interactions between breeding population, gender and age. Greater covert hue and chroma were modelled together with the appropriate tail colour measure, which was included in the models to control for within individual ability to access and deposit carotenoid pigments as tail colour is developed on shared breeding ground and therefore primarily reflects individual quality. We then progressively removed non-significant interaction terms and main effects until only significant terms remained. We then asked whether adding δ^13^C and δ^15^N values to the model explained any additional variance in greater covert colour (hue or chroma). Significance was again assessed using an F-test associated with the effect of dropping terms from a more complex model including all significant terms and the term of interest.

Finally we examined how the colour, either hue or chroma, of the tail feather and greater coverts influenced male arrival date, the clutch initiation date, colour and age of an individual’s mate, and the annual productivity of male yellow warblers in Revelstoke and Inuvik. Models examining arrival dates controlled for any variation associated with year or age before examining the effect of tail colour alone or tail plus greater covert colour. Models that included both tail and greater covert colour allowed us to assess the importance of any winter habitat effects that influenced greater covert colour, while controlling for individual quality using tail feather colour. Models examining when males acquired a mate (estimated using the clutch initiation date), as well as the age, and the colour of the mate (greater covert hue and chroma) controlled for arrival effects before examining the effect of tail colour alone or tail plus greater covert colour. Models examining annual productivity controlled for any variation associated with year, timing of breeding (clutch initiation date), age and attributes of the mate (age, hue and chroma), before examining the effects of tail colour alone or tail and greater covert colour. Models therefore controlled for any upstream effects of plumage colour and only examined direct effects of plumage colour on aspects of breeding performance. In all of these analyses, models examined the independent effects of either hue or chroma.

We used general linear models to examine factors influencing arrival dates, clutch initiation dates, and the colour of the mate acquired. We used a generalized linear model with a binomial distribution to examine factors influencing the age of the mate acquired (0-SY, 1-ASY). In these models significance of the tail and greater covert colour terms was assessed by comparing the fit of models with and without these terms using likelihood ratio tests. We used zero-altered Poisson (ZAP) models [38] to examine factors influencing the annual productivity of male yellow warblers in Revelstoke and Inuvik. ZAP models simultaneously model the effect of explanatory variables on the failure/success of individuals (0/1) and the number of young fledged if individuals are successful. These models are well suited to the analysis of productivity data where a large proportion of individuals fail to raise any young and productivity cannot be described by a normal, poison or negative binomial distribution (e.g. [39]). In these models significance of the tail and greater covert colour terms was again assessed by comparing the fit of models with and without these terms using likelihood ratio tests. An additional file presents the data set comprised of breeding data, isotope signatures and feather colour scores used in analyses [see Additional file 2]. All analyses were conducted in R 2.15.1 (The R foundation for Statistical Computing 2012).

## Results

### Population plumage characteristics

The chroma of an individual’s basic (tail) and alternate plumage (greater covert) were correlated (r = 0.68, p < 0.001, n = 357), as were the hue of an individual’s basic and alternate plumage (r = 0.51, p < 0.001, n = 356). Within feather blocks, the hue and chroma values of tail feathers were correlated (r = 0.52, p < 0.001, n = 356), as were the hue and chroma values of greater covert feathers (r = 0.67, p < 0.001, n = 362).

Yellow warblers in Revelstoke were more colourful than yellow warblers in Inuvik (Table 1 and Table 2); birds in Revelstoke had tail feathers and greater coverts with higher chroma and hue. Male yellow warblers also had tail feathers and greater coverts with higher chroma and hue than females. Older birds were generally more colourful than yearling birds. Older males had tail feathers with higher chroma values than yearling males, and all older birds had greater coverts with higher chroma values than yearling birds. Old birds also had tail feathers with higher hue than yearling birds, and older birds from Revelstoke birds had greater coverts with higher hue than yearling birds from Revelstoke.

**Table 1:** **Site**, **sex and age specific colouration of yellow warbler plumage**

		**Revelstoke**							
		**Male**				**Female**			
		**SY (n = 16)**		**ASY (n = 50)**		**SY (n = 28)**		**ASY (n = 25)**	
		**Mean**	**SD**	**Mean**	**SD**	**Mean**	**SD**	**Mean**	**SD**
Tail	Hue	53.81	0.93	54.14	0.77	53.02	1.21	53.91	0.99
	Chroma	62.12	6.71	67.62	5.46	53.99	6.61	54.72	8.1
GC	Hue	52.39	1.12	52.77	0.89	50.23	1.64	51.74	0.82
	Chroma	62.97	9.53	68.99	5.59	46.03	11.2	50.47	7.65
		**Inuvik**							
		**Male**				**Female**			
		**SY (n = 13)**		**ASY (n = 117)**		**SY (n = 52)**		**ASY (n = 62)**	
Tail	Hue	53.23	0.46	53.13	0.98	52.01	1.31	52.36	1.62
	Chroma	55.43	6.37	60.44	6.44	48.14	6.97	49.38	7.82
GC	Hue	52.82	0.58	52.58	0.87	50.96	1.06	50.96	1.15
	Chroma	60.2	7.53	65.36	6.65	46.63	7.24	48.37	8.95

Variation in colour (hue and chroma values) of the yellow inner web of the third outer right tail rectrix and yellow edge of the greater covert (GC) collected from yellow warblers breeding in Revelstoke, B.C., and Inuvik, N.W.T. Means are presented ± 1 SD.

**Table 2:** **Breeding location**, **sex and age impacts on tail and greater covert colour**

**Feather**	**Variable**	**r**^**2**^	**Model**	**β**	**SE**	**F**	**P**
Tail	Hue	0.25	**Intercept**	**52.42**	**0.12**		
			**Location**_**Revelstoke**_	**1.09**	**0.13**	**70.83**	**<0.001**
			**Sex**_**male**_	**0.71**	**0.13**	**29.49**	**<0.001**
			**Age**_**SY**_	-**0.37**	**0.14**	**6.8**	**0.009**
			Location*Sex	-	-	1.12	0.29
			Location*Age	-	-	0.8	0.37
			Sex*Age	-	-	1.44	0.23
Tail	Chroma	0.48	**Intercept**	**49.08**	**0.77**		
			**Location**_**Revelstoke**_	**6.42**	**0.77**	**69.38**	**<0.001**
			**Sex**_**male**_	**11.49**	**0.91**	-	**<0.001**
			**Age**_**SY**_	-**1.14**	**1.06**	-	-
			Location*Sex	-	-	0.92	0.34
			Location*Age	-	-	0.09	0.76
			**Sex**:**Age**	-**3.95**	**1.73**	**5.19**	**0.02**
GC	Hue	0.42	**Intercept**	**51.07**	**0.12**		
			**Location**_**Revelstoke**_	**0.39**	**0.14**	-	-
			**Sex**_**male**_	**1.45**	**0.14**	-	-
			**Age**_**SY**_	-**0.14**	**0.18**	-	-
			Location*Sex	-	-	1.69	0.19
			Location*Age	-**1.03**	**0.25**	**17.03**	**<0.001**
			Sex*Age	**0.56**	**0.27**	**4.43**	**0.04**
GC	Chroma	0.59	**Intercept**	**48.82**	**0.78**		
			**Location**_**Revelstoke**_	**2.12**	**0.87**	**6.01**	**0.01**
			**Sex**_**male**_	**16.75**	**0.87**	**372**	**<0.001**
			**Age**_**SY**_	-**3.64**	**0.95**	**14.72**	**<0.001**
			Location*Sex	-	-	1.94	0.16
			Location*Age	-	-	2.74	0.1
			Sex*Age	-	-	1.75	0.19

Summary of the effects of breeding location, sex and age on the colour (hue and chroma) of the yellow inner web of the third outer right tail rectrix and yellow edge of the greater covert (GC) collected from yellow warblers breeding in Revelstoke, B.C., and Inuvik, N.W.T. Beta values ± standard error are given for significant terms (in bold). The adjusted r^2^ is presented for the model including only significant terms.

### Basic plumage colour and winter habitat use

The chroma and hue values of an individual’s tail were unrelated to the δ^13^C or δ^15^N signatures of their greater coverts (Table 3). δ^13^C signatures varied with year and differed among populations. Birds from had greater coverts with, on average, more depleted δ^13^C signatures than birds from Revelstoke (LS means ± SE; Inuvik = -23.22 ± 0.13; Revelstoke = -21.86 ± 0.17). Males also tended to have more depleted δ^13^C signatures than females (Males = -22.67 ± 0.14; Females = -22.38 ± 0.15) but δ^13^C signatures did not vary with age. δ^15^N signatures varied with year and sex, but did not differ among populations or vary with age. Males had greater coverts with more enriched δ^15^N signatures than females (Males = 8.82 ± 0.11; Females = 8.45 ± 0.12) (Table 3).

**Table 3:** **Impacts of breeding location**, **sex**, **age and tail colour on wintering habitat acquisition**

**Winter Habitat**	**r**^**2**^	**Variable**	**β**	**SE**	**F**	**Prob** > **F**
δ^13^C	0.1	**Intercept**	-**22.88**	**0.2**	-	-
		**Year**_**2010**_	-**0.25**	**0.25**	**3.01**	**0.05**
		**Year**_**2011**_	-**0.67**	**0.28**	-	-
		**Location**_**REVELSTOKE**_	**1.33**	**0.23**	**34.03**	**<0.001**
		Sex_MALE_	-	-	3	0.08
		Age_SY_	-	-	0.13	0.72
		Tail Hue	-	-	0.2	0.66
		Tail Chroma	-	-	0.52	0.47
δ^15^N	0.02	**Intercept**	**8.67**	**0.17**	-	-
		**Year**_**2010**_	-**0.03**	**0.19**	**3.36**	**0.04**
		**Year**_**2011**_	-**0.5**	**0.21**	-	-
		Location_REVELSTOKE_	-	-	0	0.96
		**Sex**_**MALE**_	**0.33**	**0.17**	**3.9**	**0.05**
		Age_SY_			0.18	0.67
		Tail Hue			0.51	0.48
		Tail Chroma			0.32	0.57

Summary of the effects of breeding location, sex, age and tail colour (Hue and Chroma) on δ^13^C and δ^15^N ratios measured in winter grown greater coverts. We present the adjusted r^2^ for the final model. Beta values ± standard error are given for significant terms (in bold). Two-way interactions were examined but none were found to be significant (all p > 0.05).

### Winter habitat use and alternate plumage colour

δ^13^C signatures from an individual’s greater coverts were not related to the chroma or hue of the yellow edges of their greater coverts, after controlling for variation in chroma and hue associated with breeding population, sex, age and tail colour (Table 4). These results are not altered if we do not control for variation in chroma or hue of the tail (GC Chroma: δ^13^C, F = 0.49, p = 0.48; GC Hue: δ^13^C, F = 0.54, p = 0.46).

**Table 4:** **Impacts of breeding location**, **sex**, **age**, **tail colour and winter habitat on greater covert colour**

**Feather**	**Variable**	**r**^**2**^	**Model**	**β**	**SE**	**F**	**Prob** > **F**
GC	Hue	0.55	Intercept	36.6	2.78		
			**Location**_**Revelstoke**_	-**12.23**	**5.54**	-	**0.03**
			**Sex**_**male**_	**1.29**	**0.11**	-	**<0.001**
			**Age**_**SY**_	-**13.13**	**4.7**	-	**0.006**
			**Tail Hue**	**0.28**	**0.05**	-	**<0.001**
			Location*Age	-**0.91**	**0.26**	**13.15**	**<0.001**
			Age*Tail Hue	**0.25**	**0.09**	**8.39**	**0.005**
			Location*Tail Hue	**0.23**	**0.1**	**4.62**	**0.03**
			δ^13^C	-	-	0.77	0.38
			δ^15^N	-	-	2.11	0.15
GC	Chroma	0.65	Intercept	22.31	3.32		
			Location_Revelstoke_	-	-	0.83	0.36
			**Sex**_**male**_	**12**	**0.99**	**152.2**	**<0.001**
			**Age**_**SY**_	-**2.65**	**0.88**	**8.64**	**0.004**
			**Tail Chroma**	**0.42**	**0.05**	**68.73**	**<0.001**
			δ^13^C	-	-	0.06	0.81
			**δ**^**15**^**N**	**0.65**	**0.24**	**7.43**	**0.007**

Summary of the effects of breeding location, sex, age, tail colour, δ^13^C and δ^15^N on greater covert (GC) hue and chroma. We present the adjusted r^2^ for the final model. Beta values are ± standard error are given for significant terms (in bold). All two way interactions were examined but we present only those that were found to be significant (p < 0.05).

**Figure 2:**
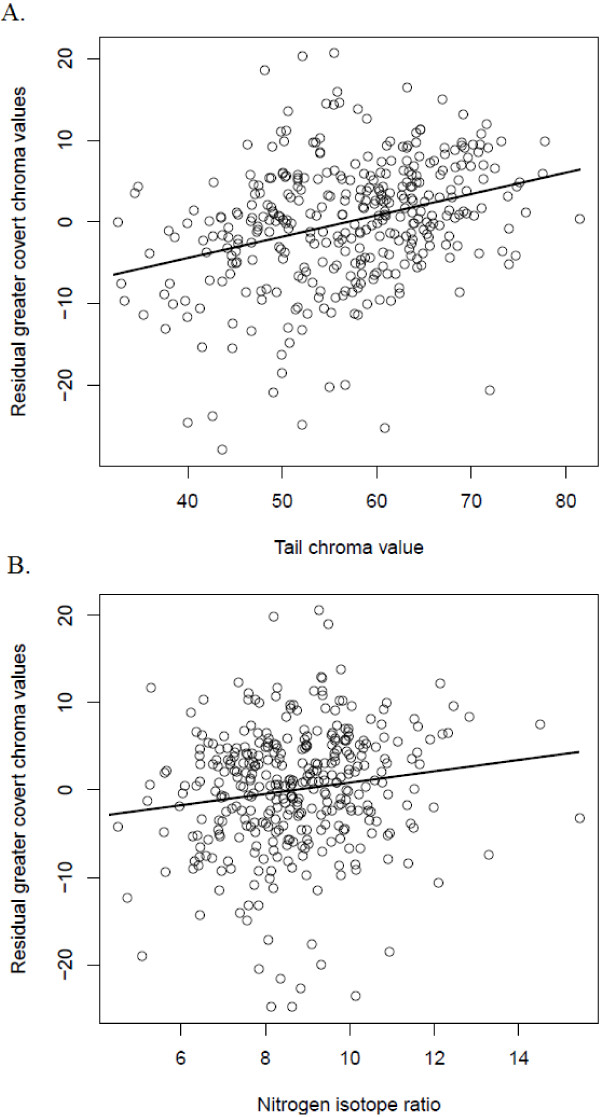
**Effects of tail chroma and nitrogen ratios on greater covert colour values.** Relationship between **A)** Tail chroma values and residual greater covert chroma values that control for the effects of sex, age and nitrogen isotope ratios in greater covert feathers, and **B)** Nitrogen isotope ratios in greater covert feathers and residual greater covert chroma values that control for the effects of sex, age and tail chroma values.

Individuals with greater coverts with more enriched δ^15^N signatures had greater coverts with higher chroma values, even after controlling for variation in chroma associated with breeding population, sex, age and tail colour (Figure 2, Table 4). δ^15^N signatures were not related to the hue of an individual’s greater coverts after controlling for variation in hue associated with breeding population, sex, age and tail colour (Table 4). These results are not altered if we do not control for variation in chroma or hue of the tail (GC Chroma: δ^15^N, F = 8.78, p = 0.003; GC Hue: δ^15^N, F = 0.45, p = 0.50).

### Plumage colour and breeding performance in Revelstoke

**Figure 3:**
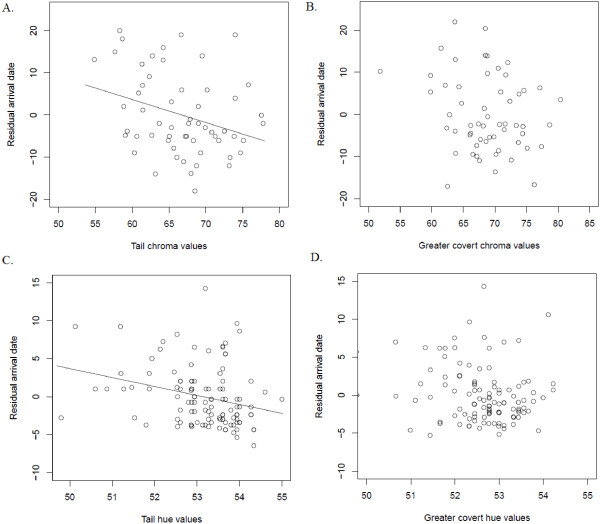
**Effects of tail and greater covert colour on male arrival dates.** Relationships between **A)** Tail chroma values and residual arrival date (controlling for age and year) for male yellow warblers in Revelstoke. **B)** Greater covert chroma values and residual arrival date (age, year and tail chroma) for Revelstoke. **C)** Tail hue values and residual arrival date (age, year and age*year interaction) for males in Inuvik and **D)** Greater covert hue values and residual arrival (age, year, age*year interaction and tail hue) for Inuvik. Lines of best fit included when significant (p > 0.05).

After controlling for year-to-year variation in male arrival dates and the earlier arrival of older males, males with higher tail chroma arrived earlier than individuals with lower chroma values (Figure 3). In contrast, tail feather hue values were unrelated to male arrival dates. After controlling for tail colour, neither greater covert chroma nor hue explained any additional variation in male arrival dates (Table 5). However, it is important to note that we would have detected a link between greater covert chroma and arrival dates had we not controlled for tail chroma in the model (GC Chroma, F = 4.5, p = 0.04). Male arrival date influenced mate acquisition: early arriving males obtained mates that initiated breeding earlier, were older and had greater coverts with higher hue values. Tail colour also had a direct effect on mate acquisition; males with higher tail hue obtained mates that were older and had higher greater covert chroma and hue values. After controlling for arrival date and tail colour, male greater covert chroma and hue values did not explain any additional variation in the clutch initiation date, age or colour of their mate (Table 5). Males that obtained a mate that initiated breeding earlier had higher annual productivity, because breeding early increased the probability of producing at least one fledgling over the course of the breeding season. Male tail feather and greater covert colour (chroma and hue) had no direct effect on annual productivity of males in Revelstoke (Table 6).

**Table 5:** Effects of plumage colour on male yellow warbler breeding phenology

**Term**	**β**	**SE**	**F**	**P**	**Term**	**β**	**SE**	**F**	**P**
**Revelstoke**					**Inuvik**				
Arrival					Arrival				
Year_2010_	-7.27	2.78	4.83	**0.02**		-	-	-	-
Year_2011_	0.85	3.15				-	-	-	-
Age_SY_	1.18	2.85	3.27	0.07		-	-	-	-
Year_2010*_Age	-	-	-	-		2.89	3.27	3.81	**0.02**
Year_2011*_Age	-	-	-	-		-5.81	3.05		
Tail chroma	-0.73	0.23	10.02	**0.002**		-	-	2.48	0.12
GC chroma	-	-	2.32	0.13		-	-	0.11	0.74
Tail hue	-	-	1.68	0.2		-1.19	0.45	6.17	**0.01**
GC hue	-	-	1.67	0.2		-	-	1.36	0.25
Clutch initiation					Clutch Initiation				
Year_2010_	3.98	1.17	4.54	**0.01**		-	-	-	-
Year_2011_	1.5	1.39				-	-	-	-
Arrival	0.53	0.07	47.3	<**0.001**		-	-	-	-
Year_2010_*Arrival	-	-	-	-		-0.2	0.31	3.01	**0.05**
Year_2011_*Arrival	-	-	-	-		-0.6	0.29		
Age	-	-	3.09	0.08		-	-	0.32	0.57
Tail chroma	-	-	0.94	0.34		-	-	0.12	0.73
GC chroma	-	-	0.83	0.37		-	-	0.88	0.35
Tail hue	-	-	0.01	0.92		-	-	0	0.99
GC hue	-	-	0	0.96		-	-	0.18	0.67
Mate hue					Mate Hue				
Arrival	-0.05	0.02	5.63	**0.02**		-	-	0.19	0.66
Age	-	-	1.94	0.17		-	-	1.54	0.22
Tail chroma	-	-	3.03	0.09		-	-	0.03	0.86
GC chroma	-	-	3.14	0.09		-	-	0.07	0.79
Tail hue	1.23	0.22	31.07	<**0.001**		-	-	0	0.99
GC hue	-	-	0.22	0.64		-	-	0.03	0.86
Mate chroma					Mate Chroma				
Arrival	-	-	0.16	0.69		-0.39	0.19	4.38	**0.04**
Age	-	-	1.18	0.28		-	-	0.35	0.56
Tail chroma	-	-	0.48	0.49		-	-	0.99	0.33
GC chroma	-	-	0.07	0.79		-	-	0.02	0.89
Tail hue	6.36	1.71	18.91	<**0.001**		-	-	0.96	0.33
GC hue	-	-	0.04	0.84		-	-	1.88	0.17
Mate age					Mate Age				
Arrival	-0.08	0.03	7.59	**0.006**		-	-	0.28	0.6
Age	-	-	0.61	0.43		-	-	0.17	0.68
Tail chroma	-	-	0.03	0.86		-	-	0.05	0.82
GC chroma	-	-	0.09	0.76		-	-	1.25	0.26
Tail hue	0.9	0.46	4.68	**0.03**		-	-	0.22	0.64
GC hue	-	-	0.03	0.87		-	-	0.59	0.44

Summary of the effects of basic and alternate plumage colour (Tail and Greater Covert Hue and Chroma) on male arrival date, clutch initiation date, mate colour and age for male yellow warblers breeding in Revelstoke, BC and Inuvik, NWT. Beta values ± standard error are given for significant terms (in bold). All two-way interactions were examined but we present only those that were found to be significant (p < 0.05). Basic plumage colour effects are examined after controlling for other significant terms in the model. Alternate plumage effects are examined after also controlling for basic plumage colour. Hue and Chroma effects are examined independently.

**Table 6:** **Effects of male age**, **breeding phenology**, **plumage colour and mate characteristics on reproductive success**

	**0**/**1**		**Count**		**0**/**1**		**Count**	
	**Revelstoke**				**Inuvik**			
	**Chi**	**p**	**Chi**	**p**	**Chi**	**p**	**Chi**	**p**
Year	2.53	0.28	0.43	0.51	7.06	**0.03**	0.54	0.76
Clutch initiation	7.71	**0.005**	0.07	0.79	0.30	0.59	0.02	0.89
Age	0.08	0.77	0.40	0.53	0.10	0.75	0.00	0.99
Tail chroma	0.89	0.35	0.19	0.66	0.24	0.62	0.00	0.97
GC chroma	0.43	0.51	2.45	0.12	2.19	0.14	0.08	0.78
Tail hue	1.59	0.21	1.01	0.31	0.02	0.89	0.05	0.82
GC hue	0.49	0.48	0.05	0.83	0.32	0.57	0.01	0.93
Mate age	0.05	0.83	0.76	0.38	2.47	0.12	0.07	0.79
Mate chroma	1.51	0.22	0.05	0.83	0.10	0.79	0.39	0.53
Mate hue	0.04	0.84	0.32	0.57	0.14	0.71	2.36	0.12

Summary of the effects of basic and alternate plumage colour (tail and greater cover hue and chroma), breeding phenology and mate quality on reproductive success of male yellow warblers breeding in Revelstoke, BC and Inuvik, NWT. The ZAP model simultaneously examines the effect of explanatory variables on the failure/success of individuals (0/1) and the number of young fledged if individuals are successful (Count). Significant terms are indicated in bold.

### Plumage colour and breeding performance in Inuvik

After controlling for the earlier arrival of older males in some years, males with higher tail hue arrived earlier than individuals with lower hue values (Figure 3). In contrast, tail feather chroma values were unrelated to male arrival dates. After controlling for tail colour, greater covert chroma and hue did not explain any additional variation in male arrival dates (Table 5). Again, had we not controlled for tail hue, it would appear that increased greater covert hue was associated with earlier arrival dates (GC Hue, F = 3.99, p = 0.05). Male arrival date influenced mate acquisition; early arriving males obtained mates that initiated clutches earlier in some years, and had greater coverts with higher chroma (Table 5). Annual productivity was lower in 2011 than 2010 and 2009, but did not vary with female clutch initiation date, age or colour. Male tail feather and greater covert colour (chroma and hue) had no direct effects on the clutch initiation date, age or colour of their mate, or the annual productivity of males in Inuvik (Table 6).

## Discussion

Carry-over effects may be driven by both extrinsic factors and the intrinsic quality of individuals, and isolating these factors is difficult in wild populations [1]. In this study, we attempted to separate the role of winter habitat carry-over effects and the role of intrinsic quality on plumage characteristics that are associated with breeding events by using two feather groups, moulted at different points in the annual cycle. This enabled us to partially control for individual factors (“quality”) that might influence colouration. With this approach, we found only limited evidence that winter habitat quality was linked to alternate plumage colouration, and no evidence that this winter grown plumage explained any variation in breeding phenology, mate acquisition, or reproductive success. Basic colouration was associated with male arrival date and some aspects of mate quality, suggesting that intrinsic factors and not a wintering to breeding season carry-over effect influenced individual productivity among male yellow warblers in temperate and arctic Canada.

Sexually selected plumage characteristics, although generally considered a reproductive trait, may also be utilized in conspecific competition for resources and/or access to wintering territories (e.g. [40,41]). Previous work with American redstarts indicate that both yearling and adult males with brighter tail feathers hold wetter (higher quality) wintering territories and that this relationship is detectible in the δ^13^C signatures of claw samples collected on the breeding grounds [41]. In contrast, we found no evidence that the colour of summer-moulted tail feathers was correlated with either δ^13^C or δ^15^N signatures of winter grown feathers in yellow warblers. Yellow warblers from Inuvik had greater coverts with more depleted δ^13^C signatures than yellow warblers from Revelstoke suggesting they occupy wetter (potentially higher quality) territories on the wintering grounds. This would be consistent with Newton’s [42] hypothesis that northern populations may arrive on wintering grounds earlier, and be more likely to occupy high quality habitat, because they finish breeding before southern populations. Male yellow warblers had more enriched δ^15^N signatures than females, which could be consistent with geographic sex-segregation on the wintering grounds [43,44].

Carotenoid based plumage colouration may reflect environmental carotenoid availability (e.g. [14,17]), but could also represent an individual’s genetic or phenotypic quality and their ability to consume, modify, transport and/or deposit carotenoids. Within-individual consistency in plumage colouration has been reported in captive goldfinches (*Carduelis tristis*) assessed before and after being fed carotenoid-rich diets [45], suggesting that carotenoid colour is influenced by factors other than the external environment. In our study, basic and alternate plumage colour was correlated, indicating that intrinsic factors likely contribute to carotenoid colour in yellow warblers.

Access to resources on high quality winter habitat can be linked to the condition of neotropical migrants throughout the overwinter period [3,6] and could also be expected to be associated with the colour of carotenoid-based winter grown plumage. However, alternate plumage colour did not vary with the δ^13^C signatures of greater covert feathers of yellow warblers from either Revelstoke or Inuvik. Similarly, Lindsay [46] found no relationship between the “yellow-ness” (hue) and δ^13^C signatures of winter-grown plumage of yellow warblers in Ohio. The available data therefore suggest that moisture levels in winter habitat are not linked to the colour of winter plumage in yellow warblers. In contrast, greater covert chroma values did vary with the δ^15^N signatures of greater covert feathers in yellow warblers in Revelstoke and Inuvik (Figure 2). Since δ^15^N signatures of blood samples collected from yellow warblers on the Pacific coast are more enriched than those collected from yellow warblers in the Gulf region of Mexico [10] access to dietary carotenoids may vary across the yellow warbler winter range. The latter result suggests one possible pathway by which winter habitat quality could influence sexually selected plumage and carry-over to influence breeding phenology and reproductive success in yellow warblers.

The colour of sexually selected alternate plumage may be associated with breeding phenology and subsequent reproductive success because more colourful males acquire a mate more rapidly (e.g. [47]), or pair with older and/or more colourful females (e.g. [48]). In this study, the colour of the basic plumage grown on the breeding grounds was correlated with male arrival on the breeding grounds the following year. In Revelstoke male arrival dates varied with tail chroma values, whereas in Inuvik male arrival dates varied with tail hue (Figure 3).

Male arrival dates were subsequently linked with the age and/or colour of their mate and the onset of breeding, which in Revelstoke determined male reproductive success. In Revelstoke, tail colour (hue) also had direct links to the age and colour of the mate the male acquired. Male plumage colour was therefore associated with variation in the timing of breeding and mate acquisition in both populations. However, the colour of the winter grown alternate plumage explained no additional variation in breeding phenology, mate acquisition, or reproductive success in either population, suggesting that winter habitat use that was associated with greater covert chroma values did not carry-over to influence reproduction.

As plumage colouration is used in sexual signalling we predicted that plumage colour would have direct links to male productivity, either because more colourful males are higher quality individuals and provide more parental care [49], or because females paired with higher quality mates invest more in offspring than those paired with less colourful males [50]. We found no evidence that male plumage colour was associated with productivity in either of our studied populations. However in our study, productivity was defined as the number of young fledged over the course of the breeding season. Plumage colouration may additionally be linked to fledgling quality and number of recruits produced; as seen in blue tits (*Cyanistes caeruleus*) in which males with higher hue values have been shown to produce fledglings in better condition [51] and the probability of fledgling recruitment has been shown to increase with maternal age and female brightness [52]. Plumage characteristics may also have direct effects on productivity through extra-pair paternity if plumage influences access to extra-pair copulations and the likely-hood of cuckoldry as previously observed in amount of breast streaking in yellow warblers [53] and in plumage colouration in American redstarts [54]. As we were interested in habitat influences on colour we limited our study of yellow warblers to the environmentally acquired carotenoid based colouration, and did not examine plumage streaking in these populations.

## Conclusions

Carry-over effects between seasons are likely due to a complex interaction between intrinsic factors related to the individual and extrinsic factors [1]. Previous studies have detected effects of winter habitat quality on over-winter condition, timing of migration and breeding success [3,6,55]. This study suggests that these carry-over effects could be driven principally by the intrinsic quality of individuals. This study therefore highlights the importance of isolating intrinsic quality when assessing potential carry-over effects, and emphasizes the need for experiments, such as removal/upgrade experiments [4], that can further disentangle the role of intrinsic and extrinsic factors on carry-over effects. Such experiments would also be necessary in examining the potential for habitat-based carry-over effects that could arise due to loss or change in winter habitat quality which could result, for example, in high-quality individuals being forced to winter in low-quality remaining habitat.

### Availability of supporting data

The data set supporting the results of this article is included within the article in Additional file 2.

## Competing interests

The authors declare that they have no competing interests.

## Authors’ contributions

DJG and AD designed the study. AD and TBJ collected the feather samples and reproductive data. TBJ and DJG performed the statistical analysis. TBJ performed the colour analysis and drafted the manuscript; TBJ, AD and DJG edited the manuscript. All authors have read and approved the final manuscript.

## Supplementary Material

Additional file 1 **Hue and Chroma chart.** Chart showing changes in colour with increasing values of hue and chroma. This chart does not consider other colour properties (e.g. brightness) but represents how a yellow swatch changes with differing hue and chroma values. Click here for file

Additional file 2 **Breeding, isotope and colour score dataset.** Excel spread sheet of data set used in analyses. Includes individual’s greater covert isotope signatures, feather hue and chroma scores, and breeding attributes (arrival date, clutch imitation date, number of fledglings, mate age and mate colour). Click here for file
