# Supplementary figures and images for: Individual quality explains association between plumage colouration, arrival dates and mate acquisition in yellow warblers (Setophaga petechia)
Source: BMC Ecol. 2014 May 7;14:13. doi: 10.1186/1472-6785-14-13 (PMC4024118; doi:10.1186/1472-6785-14-13)

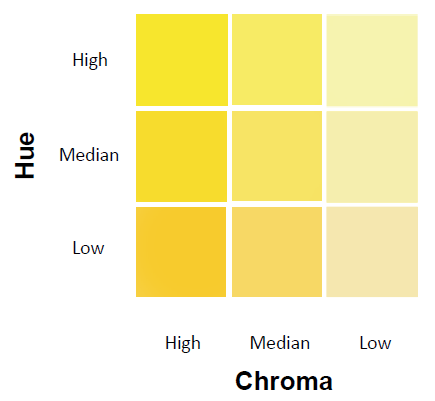

Supplement: Additional file 1 — Hue and Chroma chart. Chart showing changes in colour with increasing values of hue and chroma. This chart does not consider other colour properties (e.g. brightness) but represents how a yellow swatch changes with differing hue and chroma values. [file 1472-6785-14-13-S1.png]
